# Comparison of the bacterial community composition in the granular and the suspended phase of sequencing batch reactors

**DOI:** 10.1186/s13568-017-0471-5

**Published:** 2017-09-05

**Authors:** Enikö Szabó, Raquel Liébana, Malte Hermansson, Oskar Modin, Frank Persson, Britt-Marie Wilén

**Affiliations:** 1 Division of Water Environment Technology, Department of Architecture and Civil Engineering, Chalmers University of Technology, 41296 Gothenburg, Sweden; 2 Department of Chemistry and Molecular Biology, University of Gothenburg, 40530 Gothenburg, Sweden

**Keywords:** Aerobic granular sludge, Microbial community composition, Wash-out dynamics, Temporal variation, Spatial distribution, Sequencing batch reactors

## Abstract

Granulation of activated sludge is an increasingly important area within the field of wastewater treatment. Granulation is usually achieved by high hydraulic selection pressure, which results in the wash-out of slow settling particles. The effect of the harsh wash-out conditions on the granular sludge ecosystem is not yet fully understood, but different bacterial groups may be affected to varying degrees. In this study, we used high-throughput amplicon sequencing to follow the community composition in granular sludge reactors for 12 weeks, both in the granular phase and the suspended phase (effluent). The microbiome of the washed out biomass was similar but not identical to the microbiome of the granular biomass. Certain taxa (e.g. *Flavobacterium* spp. and *Bdellovibrio* spp.) had significantly (p < 0.05) higher relative abundance in the granules compared to the effluent. Fluorescence in situ hybridization images indicated that these taxa were mainly located in the interior of granules and therefore protected from erosion. Other taxa (e.g. *Meganema* sp. and *Zooglea* sp.) had significantly lower relative abundance in the granules compared to the effluent, and appeared to be mainly located on the surface of granules and therefore subject to erosion. Despite being washed out, these taxa were among the most abundant members of the granular sludge communities and were likely growing fast in the reactors. The ratio between relative abundance in the granular biomass and in the effluent did not predict temporal variation of the taxa in the reactors, but it did appear to predict the spatial location of the taxa in the granules.

**Electronic supplementary material:**

The online version of this article (doi:10.1186/s13568-017-0471-5) contains supplementary material, which is available to authorized users.

## Introduction

Wastewater treatment by aerobic granular sludge is a low-footprint technology that allows effective pollutant removal even at high loading rates (Beun et al. 1999; de Bruin et al. 2004). During granulation, bacterial cells are self-immobilized in an EPS (extracellular polymeric substances) matrix, resulting in a dense, compact structure with an anaerobic/anoxic core and an aerobic outer layer (Beun et al. 2001; de Kreuk et al. 2005).

Granulation is usually achieved by high hydraulic selection pressure, i.e. a sequencing batch reactor (SBR) is operated either with short settling time (variable volume operation mode) or with high upflow velocity (constant volume operation/fill-draw mode), both resulting in the wash-out of slow settling particles. Low effluent quality due to high suspended solids (SS) concentration has frequently been reported both in laboratory-scale and pilot-scale applications, fed with synthetic and/or real wastewater (Inizan et al. 2005; Yilmaz et al. 2008; Morales et al. 2013; Rocktäschel et al. 2015; Derlon et al. 2016). Strategies to decrease the effluent SS concentration include lower upflow velocity combined with selective sludge removal (Pronk et al. 2015; Derlon et al. 2016) or longer settling time and lower degree of granulation (Rocktäschel et al. 2015).

The ecological implications of the strong wash-out conditions on the microbial community are not yet fully understood, despite being one of the strongest selective forces applied in SBRs for aerobic granulation. It has been shown that the wash-out rate of different bacterial groups can be different, depending on their spatial distribution within the granule (Winkler et al. 2012). Bacterial populations growing on the surface of the granules may be eroded and washed out in higher numbers than bacteria growing deeper in the granule. Thus, bacterial groups situated in the exterior layer of the granules are likely to contribute more to the suspended solids content in the effluent than bacteria situated in the interior layer. However, if a granule breaks up, exterior and interior bacterial populations will be washed out equally, presuming that the particles are not too dense. The density of the broken granule particle depends on the type(s) of bacteria it is comprised of (Gonzalez-Gil and Holliger 2014), and on the predominant granulation mechanisms—self-aggregation of floccular biomass, self-aggregation of small granules (microcolony aggregation), microcolony outgrowth, or attachment of floccular biomass to granular biomass (Barr et al. 2010; Verawaty et al. 2012; Zhou et al. 2014). Depending on process conditions and the bacteria dominating the microbial community, different granulation mechanisms were reported to prevail (Weissbrodt et al. 2013).

In this study, we followed the community composition in granular sludge reactors at different operational conditions for 12 weeks, both in the granular phase and the suspended phase (effluent). This work is, to our knowledge, the first report about the microbial community composition in the effluent of granular sludge reactors. We assessed how the spatial distribution of certain taxa affected its wash-out, and compared the bacterial community in the granular and suspended phases to gain better understanding of the successional patterns in granular sludge reactors.

## Materials and methods

### Reactor setup and medium

The experiments were carried out in three column-shaped SBRs, each with a working volume of 3 L, a diameter of 6 cm, and a total height of 132 cm. The influent was pumped in at the bottom of the reactor. The air was introduced also at the bottom through a diffusor stone (pore size 1 µm) with a superficial upflow air velocity of 1.5 cm/s. The effluent was withdrawn at 63 cm from the bottom, resulting in a volume exchange ratio of 43%. The reactors were run with 4 h cycles, one cycle consisted of 5 min filling, 55 min anaerobic phase, 143–171 min aerobic phase, 2–30 min settling, 5 min withdrawal and 2 min idle. The settling time was gradually decreased (Additional file 1: Figure S1) to allow a better retention of nitrifying organisms (Szabó et al. 2016), and the aerobic phase was concomitantly increased to permit an even, 4 h cycle length. The reactors were seeded from a full-scale wastewater treatment plant (Gryaab, Gothenburg) with aerobic/anoxic activated sludge. The reactors were fed with a 50–50% mixture of synthetic and real wastewater (6-times diluted reject water from the dewatering of digested sludge). The synthetic wastewater consisted of a concentrated acetate solution and an inorganic salt solution, pumped from separate bottles. The final composition of the synthetic wastewater is given in Additional file 1: Table S1. Reject water was used as the source of ammonium. The total organic and nitrogen loading rates as well as the influent COD and N concentrations are shown in Table 1. Three different organic loading rates were used, which allowed the comparison of the effect of wash-out at different food-to-microorganism ratios. The pH and the temperature of the reactors were not regulated, and varied in the range of 7.0–9.0 and 19–21 °C, respectively.

**Table 1:** Operational parameters and process performance

Parameter	Unit	R1	R2	R3
t	H	4	4	4
OLR	kg COD/m^3^/day	3.71 ± 0.04	1.87 ± 0.04	0.91 ± 0.04
NLR	kg NH_4_–N/m^3^/day	0.22 ± 0.02	0.22 ± 0.02	0.22 ± 0.02
COD:N ratio	–	100:6	100:12	100:24
Influent COD	mg/L	1416 ± 14	712 ± 14	346 ± 14
Influent NH_4_–N	mg/L	85 ± 6	85 ± 6	85 ± 6
E_COD_	%	98.3 ± 1.1	96.0 ± 1.4	86.3 ± 0.6
E_NH4_	%	100	100	100
E_TN_	%	65.5 ± 7.2	38.1 ± 16.4	0.4 ± 19.0
Effluent SS	mg/L	301 ± 98	133 ± 28	74 ± 13

Cycle length (t); organic and nitrogen loading rate (OLR, NLR); COD:N ratio; influent COD and NH_4_–N concentration; removal efficiency (E) of COD, NH_4_ and TN during the last 4 weeks; average effluent suspended solids concentration (SS)

### Sampling and chemical analysis

Effluent parameters were measured three times a week with a Shimadzu TOC analyzer (total organic carbon, total nitrogen) and a Dionex ICS-900 ion chromatograph (NH_4_–N, NO_2_–N, NO_3_–N). Total suspended solids and volatile suspended solids in the effluent were measured according to standard methods (APHA 1995). Biomass samples of 100 mL were withdrawn from the reactors three times per week.

### Process performance

The process performance reached steady state after approximately 6 weeks of operation. The COD removal was stable from the first day of operation, above 95, 90 and 80% in R1, R2 and R3 respectively. The suspended solids concentration in the effluent fluctuated between 0.04 and 0.5 g/L (Additional file 1: Figure S2). Complete ammonium removal was achieved after 3, 4 and 6 weeks in R1, R2 and R3 respectively (Additional file 1: Figure S3). The nitrite concentration peaked after 3–4 weeks of operation, and all nitrite was fully converted to nitrate after 6–7 weeks of operation. The total nitrogen removal also reached steady state after 6–7 weeks. Based on the process performance, weeks 1–6 are defined as the start-up period, and weeks 7–12 are defined as the steady state period. The average removal efficiency of organic material, ammonium and total nitrogen during the last 4 weeks are shown in Table 1.

### DNA extraction, PCR, sequencing and data analysis

Biomass for DNA extraction was collected three times per week, both from the withdrawn reactor samples (granular phase) and from the effluent (suspended phase), at the end of the aerated phase. DNA was extracted using the FastDNA Spin Kit for Soil (MP Biomedicals) following the manufacturer’s protocol, from 46 ± 12 mg of biomass (wet weight) per sample. 16S rRNA genes were amplified in duplicates, using the AccuPrime Pfx SuperMix (Life Technologies), 20 ng template, and 1 µM forward (515F) and 1 µM reverse (806R) primers, dual-indexed according to Kozich et al. (2013). The PCR reaction, carried out in a Biometra T3000 Thermocyler, started with 5 min enzyme activation at 95 °C, followed by 30 cycles of denaturation (95 °C, 20 s), annealing (50 °C, 15 s) and elongation (68 °C, 60 s), and finished by a 10 min final elongation at 68 °C. The duplicate PCR products were pooled, the DNA concentrations were normalized and the samples were purified using the Agencourt AMPure system (Beckman Coulter). The PCR products were multiplexed and diluted with Tris–Cl (pH 8.5, 0.1% Tween20) for a final concentration of 0.6 ng/μL, as measured by Qubit 2.0 (Life Technologies). The expected concentration and size of the pooled PCR product was confirmed by TapeStation 2200 (Agilent Technologies). PhiX control library was spiked in at 7.5%. Sequencing was performed on an Illumina MiSeq using the MiSeq Reagent Kit v2. In total, 78 samples were analyzed in this study, 26 from each reactor (13 from the granular phase and 13 from the effluent). The sequences were processed and classified, using the MiDAS taxonomy, as published in Albertsen et al. (2015), prior to statistical analyses in R (R Core Team 2016). Retention ratios were calculated by dividing the relative read abundance in the reactor with the relative read abundance in the effluent. Margalef’s species richness and Pielou’s evenness were calculated using the package vegan (Oksanen et al. 2015), non-metric multi-dimensional scaling (NMDS) ordination plots and heatmaps were created using the package ampvis (Albertsen et al. 2015), and basic R functions were used to create the Tukey boxplots, perform one-sample Wilcoxon signed-rank tests and calculate Pearson correlation coefficients (r). The sequences were deposited as an NCBI BioProject (BioProject ID: PRJNA384775).

### Fluorescence in situ hybridization (FISH)

FISH was performed on intact granules harvested from the reactors at day 55. Granules were fixed in 4% paraformaldehyde for 8 h at 4 °C and washed twice with PBS. Fixed granules were stored in PBS/ethanol (50:50) at −20 °C until use. For cryosectioning, granules were incubated overnight at 4 °C in O.C.T. Compound (VWR, Radnor, PA, USA) in individual plastic containers. Thereafter, a dry ice fume chamber was used to freeze each granule in blocks, which were stored at −70 °C until use. Granule cryosections, 10–20 µm thick, were obtained at −20 °C using a HM550 microtome cryostat (MICROM International GmbH, Germany). The cryosections were collected on SuperFrost^®^ Plus Gold microscope slides (Menzel GmbH, Germany) and stored at −20 °C. Prior to FISH, a Liquid Blocker Mini PAP Pen (Life Technologies) was used to apply a hydrophobic barrier on the glass slides framing the cryosections, which were subsequently covered with a thin layer of agarose (1% w/v) and dehydrated in an ethanol series (50, 80 and 96% v/v). FISH was performed at 46 °C for 2 h (Manz et al. 1992) using the probes and hybridization conditions shown in Table 2. Syto 40 was used as a counterstain. The target organisms were chosen based on their retention ratios. Confocal images were acquired using a Zeiss LSM700 (Carl Zeiss, Germany) with 10×/0.45 plan-apochromat and 40×/1.3 plan-apochromat oil objectives and laser diode lines of 405, 488, 555 and 639 nm. Large images covering the entire granules and large sections were acquired using the averaging (n = 4) and tiling functions of Zeiss ZEN2010 software.

**Table 2:** Probes and hybridization conditions for FISH

Probe	Target organism	FA (%)	References
BDE525	Genus *Bdellovibrio*	35	Mahmoud et al. (2007)
CFB563	Most *Flavobacteria*	20	Weller et al. (2000)
Meg983	*Meganema perideroedes*	35	Thomsen et al. (2006)
Meg1028	*Meganema perideroedes*	45	Thomsen et al. (2006)
*ZRA23a*	Most members of the *Zoogloea* lineage	35	Rosselló-Mora et al. (1995)
ZOGLO-1416	*Zoogloea* spp.	35	Loy et al. (2005)

*FA* formamide concentration in the hybridization buffer

## Results

### Population dynamics

**Fig. 1:**
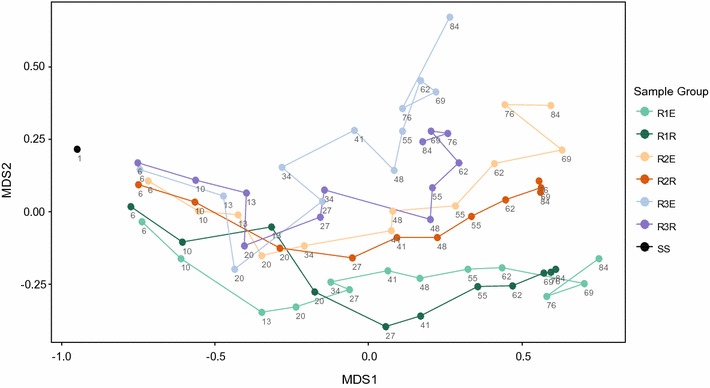
Non-metric multidimensional scaling (NMDS) ordination based on Bray–Curtis dissimilarity matrix. Samples are indicated with the number of days since seeding. R1E, R2E, R3E: effluent samples (suspended phase); R1R, R2R, R3R: samples from the reactor content (granular phase), *SS* seed sludge

The overall trend in the evolution of the bacterial communities is shown in Fig. 1. The community composition of the granular and of the suspended phase followed the same dynamics throughout the experiment in all three experimental set-ups. The average community similarity (Bray–Curtis) between contemporaneous granular and effluent samples from the same reactor is 65 ± 2% in R1, 63 ± 6% in R2, and 65 ± 7% in R3. For comparison, the average similarity between the three different experimental set-ups was 26–40% by the end of the experiment. This difference is expected, due to the different OLR applied to the reactors. The effect of the different loading rates on the microbial population dynamics is discussed in a separate publication (Szabó et al. 2017). Correlation analyses show a strong correlation between samples from the effluent and samples from the reactor content (Additional file 1: Figure S4).

### Retention ratios of abundant microorganisms

**Fig. 2:**
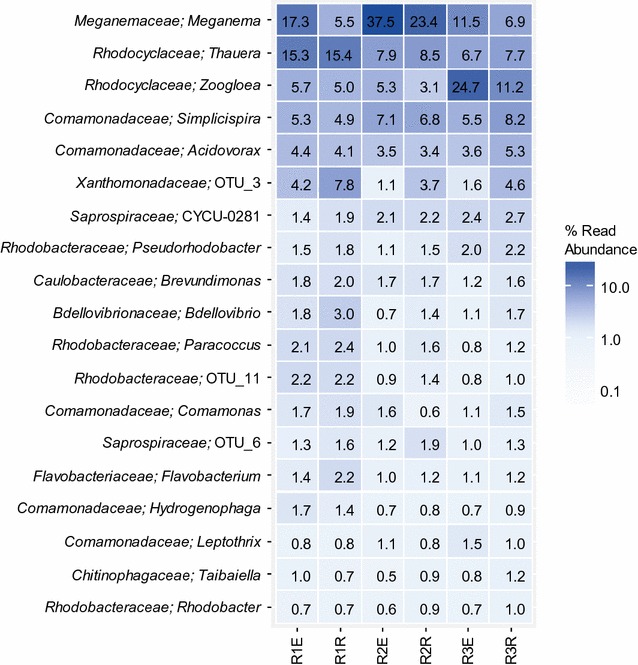
Average relative read abundance (n = 13) of the most abundant genera common for all six sample categories. These 19 genera add up to 65 ± 11% of the total population in the samples. R1E, R2E, R3E: effluent samples (suspended phase); R1R, R2R, R3R: samples from the reactor content (granular phase)

Although the community composition of the effluent and the granules was similar, it was not identical. As it can be seen in Fig. 2, there are notable differences between the relative read abundances of certain dominant genera in the suspended and granular communities.

To be able to analyze the difference between effluent and granule samples more thoroughly, the ratio of the relative abundances in contemporaneous samples was calculated for the dominant genera. This approach is similar to that suggested by Winkler et al. (2012), where they use a dimensionless “species proportion ratio” to determine whether preferential wash-out of certain bacterial groups occurs in the granular sludge reactor. We calculate the “retention ratio” by dividing the relative read abundance in the granules with the relative read abundance in the effluent. Thus, a retention ratio larger than 1 indicates good retention of the taxa in the reactor.

The correlation analysis (Table 3) of the retention ratios and the number of days since start-up show that some taxa (e.g. *Acidovorax* in R2 and R3, *Brevundimonas* in R1) were increasingly retained as the experiment progressed. Other genera became progressively more abundant in the effluent (e.g. *Meganema* in R1, *Leptothrix* in R2), although this was a less common phenomenon.

**Table 3:** Correlation analyses of retention ratios and the number of days since start-up

	R1		R2		R3	
	p	R	p	r	p	r
g__*Acidovorax*	0.8285	−0.0668	0.0099**	0.6841	0.0065**	0.7104
g__*Bdellovibrio*	0.2792	0.3584	0.0579•	0.5380	0.0143*	0.7405
g__*Brevundimonas*	0.0084**	0.6947	0.0953•	0.4821	0.0542•	0.5449
g__*Comamonas*	0.8125	−0.0731	0.0127*	0.6915	0.3800	0.2941
g__*Flavobacterium*	0.0073**	0.7033	0.0006***	0.8418	0.0596•	0.5350
g__*Hydrogenophaga*	0.0720•	0.5145	0.6511	0.1388	0.0198*	0.6346
g__*Leptothrix*	0.5783	0.1702	0.0376*	−0.5803	0.6190	0.1525
g__*Meganema*	0.0296*	−0.6524	0.1573	−0.4352	0.1005	−0.4756
g__*Paracoccus*	0.2592	0.3377	0.5587	0.1789	0.9165	0.0323
g__*Pseudorhodobacter*	0.0042**	0.7350	0.4281	0.2408	0.8144	−0.0723
g__*Rhodobacter*	0.1282	0.4443	0.4015	0.2545	0.8502	0.0612
g__*Simplicispira*	0.5592	0.1787	0.2985	0.3125	0.1308	0.4417
g__*Taibaiella*	0.5992	−0.1610	0.4869	0.3181	0.1255	0.4471
g__*Thauera*	0.4142	−0.2479	0.0008***	0.8100	0.0258*	0.6132
g__*Zoogloea*	0.4673	−0.2324	0.9779	−0.0090	0.5396	−0.1875
OTU_11	0.4770	−0.2167	0.4361	0.2368	0.3820	−0.2778
OTU_6	0.0007***	0.8571	0.0105*	0.7612	0.0185*	0.6640
OTU_3	0.2225	0.3632	0.7140	0.1331	0.0049**	0.7767
g__CYCU.0281	0.5169	0.1979	0.0103*	0.6818	0.0677*	0.5437

Significant correlations are marked with asterisk(s). Cutoff levels: (•) p < 0.1, (*) p < 0.05, (**) p < 0.01, (***) p < 0.001. For OTU_3, OTU_6 and OTU_11, the closest matches by BLASTn (Altschul et al. 1990) are shown in Additional file 1: Table S2

**Fig. 3:**
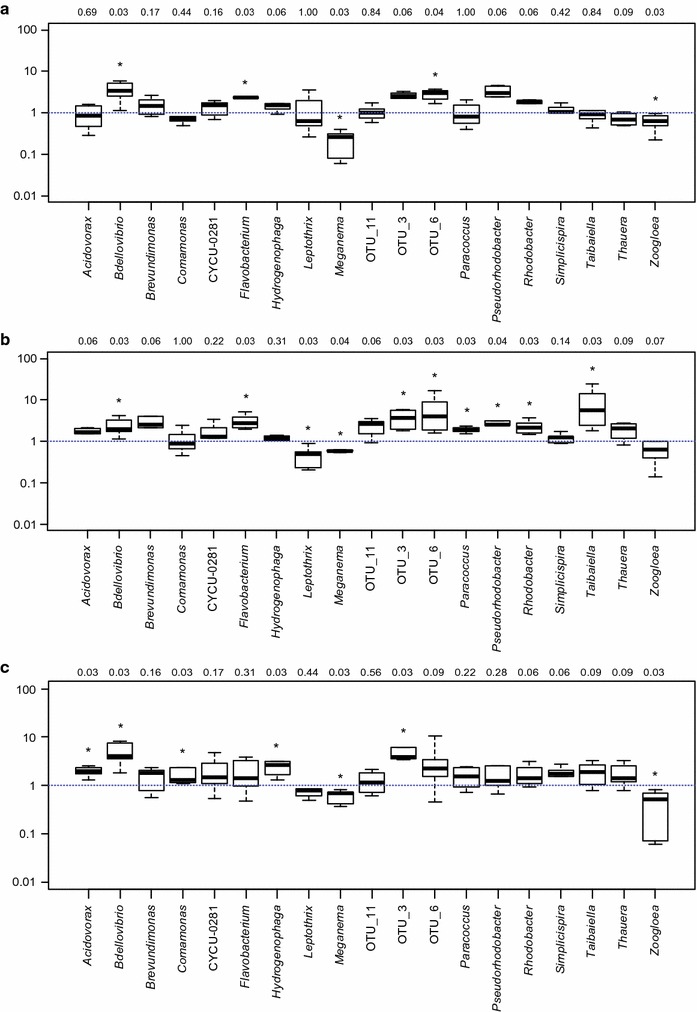
Boxplots of the retention ratios during steady-state operation (weeks 7–12) in R1 (**a**), R2 (**b**) and R3 (**c**). Values significantly different from 1 are marked with *asterisk* (p values are shown *above the plots*)

In many cases, the retention ratio did not show any significant temporal trends. However, for some genera, statistically significant differences between the start-up phase (weeks 1–6) and the steady-state operation (weeks 7–12) could be found (Fig. 3). During start-up (the first 6 weeks of operation), most genera had a retention ratio around 1, i.e. they were washed out proportionally to their relative abundance in the granular biomass (Additional file 1: Figure S5); while during steady-state operation, the retention ratio of certain genera was significantly higher (or lower) than one (Fig. 3). The average relative read abundances of the most common genera for the start-up and steady-state period are shown in Additional file 1: Figure S6.

### FISH on granule cryosections

To assess the spatial localization of bacteria with high or low retention ratios, cryosectioned slices of granules were dyed with FISH probes targeting some of the most abundant genera.

**Fig. 4:**
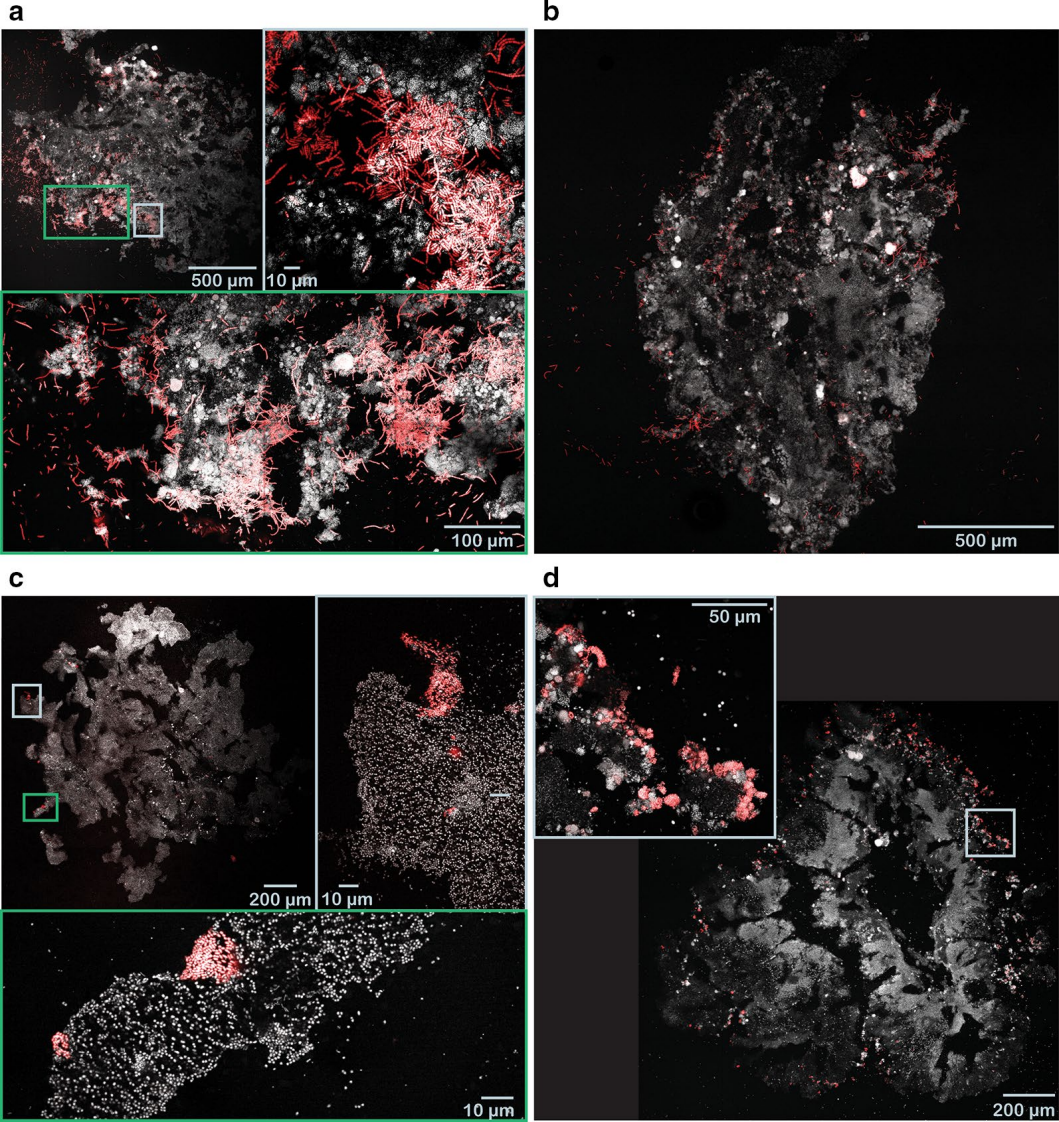
FISH-CLSM images of selected bacteria with retention ratios significantly lower than one. Cryosections of granules at ×200 magnification and detailed sections at ×400 magnification. **a**, **b** *Meganema perideroedes* in granules from R2; **c** *Zoogloea* spp. in granules from R2; **d** *Zoogloea* spp. in aerobic granules from R3. *Grey* total cells (Syto 40); *red Meganema perideroedes* (**a**, **b**) and *Zoogloea* spp. (**c**, **d**)

*Meganema* and *Zoogloea* are genera that had retention ratios significantly lower than one during steady-state operation. Both genera were found to be growing mainly on the surface of the granules (Fig. 4). The filaments of *Meganema* extend outside the granule surface. *Zoogloea* was found exclusively within 100 µm from the surface; the outgrowths resemble the finger-like structures typical for *Zoogloea*.

**Fig. 5:**
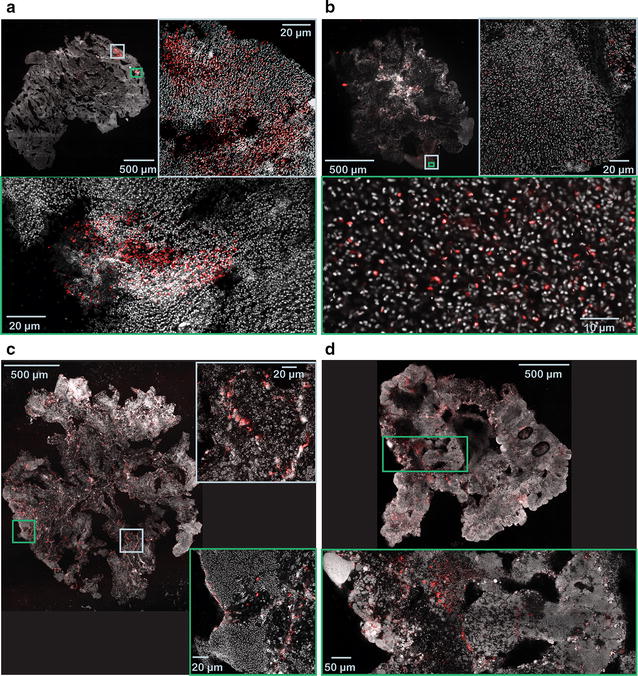
FISH-CLSM images of selected bacteria with retention ratios significantly higher than one. Cryosections of granules at ×200 magnification and detailed sections at ×400 magnification. **a**, **b** Genus *Bdellovibrio* in granules from R1; **c**, **d** *Flavobacterium* spp. in granules from R1. *Grey* total cells (Syto 40); *red* genus *Bdellovibrio* (**a**, **b**) and *Flavobacterium* spp. (**c**, **d**)

*Bdellovibrio* and *Flavobacterium* are two genera with retention ratios significantly higher than one during steady-state operation. They were found to be growing often in the deeper regions of the granule (Fig. 5).

## Discussion

Based on the dissimilarity matrices and the correlation analyses, it appears that the community composition of the effluent is similar but not identical to the community composition of the granular sludge. By calculating the retention ratios, the similarities and differences could be examined more thoroughly. Some genera became more abundant in the effluent as the experiment progressed. Other genera, on the contrary, were increasingly retained as the flocculated biomass turned into granules.

During start-up (the first 6 weeks of operation), many genera seemed to be washed out proportionally to their relative abundance in the biomass. We assume that during start-up a large fraction of the biomass is still flocculated, and every floc has similar settleability. Thus the probability of cells being discharged with the effluent is similar for every taxa, irrespective of which floc they are attached to or where in the floc they are situated. As soon as granules start to develop, differences in settleability emerge, and depending on the spatial location and the granulation mechanisms some cells are more likely to be washed out than others.

During steady-state operation (weeks 7–12), most of the biomass is granulated and dense enough to be retained in the reactor. Nonetheless, certain genera had a retention ratio significantly lower than one (Fig. 3), i.e. they were more abundant in the effluent than in the granules. A possible explanation is that these genera were situated on the surface of the granule, and were therefore exposed to granule erosion, as suggested by Winkler et al. (2012). They found that bacterial groups situated on the surface (e.g. ammonium oxidizing bacteria) were more prone to be washed out than bacterial groups situated in the core of the granules. We found that two abundant groups of bacteria, *Meganema* sp. and *Zoogloea* sp., which had a low retention ratio in all three reactors during steady-state operation (Fig. 3), were situated mostly in the outer layer of the granules, where the biomass was loosely packed (Fig. 4) and therefore more likely to be washed out due to granule erosion. *Meganema* sp. is usually found in aerobic environments, therefore it is not expected to grow in the inner parts of the granules (Kragelund et al. 2005). *Zoogloea* sp. has been reported to produce EPS containing high amounts of water and to grow as slimy colonies, thus the high abundance of these bacteria in the outer layer might explain the lower density of this part of the granule (Thomsen et al. 2007; Nielsen et al. 2010). Moreover, the typical morphological characteristics of these genera (filamentous growth for *Meganema* sp. (Kragelund et al. 2005), and finger-like structures in case of *Zoogloea* sp. (Rosselló-Mora et al. 1995) make them even more likely to detach when exposed to high shear force. Another possible explanation for the high abundance in the effluent is that these bacteria grew not only attached to the granule, but also in the bulk liquid as free floating cells. Both *Meganema* and *Zoogloea* have high substrate uptake rate and growth rate (Roinestad and Yall 1970; Kragelund et al. 2005), therefore they may also grow in suspended phase even in SBRs operated at 2 min settling time and 9.3 h HRT. It has also been shown that suspended biomass can be retained under wash-out conditions if attached to the rough surfaces of broken granules (Verawaty et al. 2012) or sheltered in indentations on the granule surface (Gonzalez-Gil and Holliger 2014).

Some genera had a retention ratio significantly larger than one during steady-state operation (Fig. 3). These genera had a higher relative abundance in the granules than in the effluent, and were not washed out of the reactor. However, this does not necessarily mean that their relative abundance in the granules increased with time. For example, *Brevundimonas* in R1, *Acidovorax* in R2, or *Comamonas* in R3 had an average retention ratio above one during steady-state operation (Fig. 3; Table 3), but their relative abundance decreased with time (Additional file 1: Figure S7). In general, no statistically significant correlation was found between the retention ratio and the temporal variation of relative abundance. Slow growing species located in the interior of the granule may be protected from erosion and thus have a high retention ratio (>1), but their relative abundance is likely to decrease because they become outnumbered by other species with higher growth rate. Taxa with consistently high or increasing relative abundance (Additional file 1: Figure S7) and high retention ratio (e.g. *Flavobacterium* and *Bdellovibrio* in R1, or OTU_6 in R2 and R3) are presumably growing well under the respective operational conditions, and they are probably growing in the core of the granules (protected from erosion). Indeed, *Flavobacterium* and *Bdellovibrio* were located in the inner parts of the granule (Fig. 5), thus suggesting that genera with high retention ratios are actually growing in deeper parts of the granules. *Bdellovibrio* is a bacterial predator (Rosenberg et al. 2014), while *Flavobacterium* spp. have been reported to hydrolyze various substrates (Bernardet et al. 1996), thus they are likely to grow on living or dead biomass, soluble microbial products, and EPS. These substrates can be found in the core of the granule. The presence of predatory bacteria like *Bdellovibrio* has been reported earlier in granular sludge (Wan et al. 2014; Weissbrodt et al. 2014; Li et al. 2014), but the effect of predation on the ecosystem is not yet fully understood. Despite being obligate aerobes (Rosenberg et al. 2014), *Bdellovibrio* spp. were located in the inner parts of the granule. It has been reported earlier that certain *Bdellovibrio* species can predate under anoxic conditions (Monnappa et al. 2013). Moreover, we have found that oxygen can penetrate the deeper regions of the granules through channels (Szabó et al. 2017), where most of the *Bdellovibrio* cells were found (Fig. 5).

The core of the granule is not only protected from erosion, but also provides microaerobic and anaerobic niches [above a certain diameter, i.e. after a certain number of weeks of operation, and depending on the bulk COD concentration (Szabó et al. 2017)]. Thus, denitrifying organisms (e.g. *Acidovorax* spp., *Pseudorhodobacter* spp., *Rhodobacter* spp., Fig. 3; Table 3) can be expected to have a high retention ratio (low relative read abundance in the effluent). Bacteria situated in the core are washed out only in case the granule breaks up, and due to the young age (<100 days) of our granules it is likely that very few granules broke up during the experiment (Gonzalez-Gil and Holliger 2014).

EPS producing taxa (e.g. *Meganema*, *Thauera*, and *Zoogloea*) were observed in high abundance in every reactor, which suggests that the predominant aggregation mechanism was microcolony outgrowth (Weissbrodt et al. 2013). These genera are usually present in conventional activated sludge ecosystems, but in lower abundances (in the seed sludge the cumulative relative read abundance of EPS producers was only 13%). Many of the EPS producing genera typical for sewage treatment plants are mixotrophic bacteria capable of denitrification and/or PHA production (Etchebehere et al. 2003; Lu et al. 2014; McIlroy et al. 2015; Inoue et al. 2016; McIlroy et al. 2016). EPS was earlier reported to play an important role in the formation and (mechanical) stability of granular sludge (Weber et al. 2007; Lemaire et al. 2008; Tan et al. 2014), while denitrification was reported to accelerate granule formation (Wan and Sperandio 2009; Suja et al. 2015). The EPS producing functional group was dominated by different genera in the three reactors (*Thauera* in R1, *Meganema* in R2 and *Zoogloea* in R3) due to the different organic loading rates applied.

It can be concluded that the community composition of the suspended phase is similar but not identical to the community composition of the granular phase. Complex patterns were observed for the dynamics of the reactor and effluent microbial communities. During the first part of the experiment, most genera showed similar relative abundance in the reactor and effluent samples. Once granules were developed, bacterial groups located in the interior of the granules tended to be present in lower numbers in the effluent, while taxa growing on the surface and/or in the bulk phase were more abundant in the effluent. However there was no correlation between the degree of wash-out and the temporal dynamics of individual taxa in the granules. Bacteria that were well retained in the granules may be numerically outcompeted over time, as seen by their decreasing relative abundance. On the other hand, bacteria that may appear to be washed out of the system, as seen by their high relative abundance in the suspended phase, may in fact grow in suspension and might even reattach to the granules.

## Additional file

**Additional file 1.** Additional figures and tables.

## Abbreviations

COD: chemical oxygen demand
EPS: extracellular polymeric substance
FISH: fluorescence in situ hybridization
HRT: hydraulic retention time
NMDS: non-metric multidimensional scaling
OLR: organic loading rate
OUT: operational taxonomic unit
PCR: polymerase chain reaction
SBR: sequencing batch reactor
SS: suspended solids
