# Supplementary material for: Comparison of the bacterial community composition in the granular and the suspended phase of sequencing batch reactors
Source: AMB Express. 2017 Sep 5;7:168. doi: 10.1186/s13568-017-0471-5 (PMC5583138; doi:10.1186/s13568-017-0471-5)
Supplement: Supplementary file 1 — Additional file 1. Additional figures and tables. [file 13568_2017_471_MOESM1_ESM.pdf]

Supplementary material

Comparison of the bacterial community composition in the granular and the suspended phase of sequencing batch reactors

Enikö Szabó*, Raquel Liébana, Malte Hermansson, Oskar Modin, Frank Persson, Britt-Marie Wilén

* Corresponding author. Division of Water Environment Technology, Department of Civil and Environmental Engineering, Chalmers University of Technology, SE-41296 Gothenburg, Sweden. E-mail address: eniko.szabo@chalmers.se

Table S1 Composition of the synthetic wastewater

	R1	R2	R3
$NaCH_3COO$ (mg/L)	2212.2	1058.0	457.1
CH_3COOH (mg/L)	910.5	435.5	188.3
K_2HPO_4 (mg/L)	71.0	35.5	17.8
$CaCl_2$ (mg/L)	22.1	22.1	22.1
$MgSO_4 \times 7H_2O$ (mg/L)	24.4	24.4	24.4
$FeSO_4 \times 7H_2O$ (mg/L)	19.5	19.5	19.5
Micronutrients (mL/L)[1]	1	1	1

[1] The recipe of the micronutrient solution can be found in Tay et al. [1]

Table S2 Closest matches by BLASTn [2] in the NCBI database

OTU	Closest match	Genbank accession no.	Similarity
OTU_11	*Catellibacterium aquatile*	NR_114265.1, NR_116272.1	100%
	Gemmobacter fontiphilus	NR_108454.1	100%
OTU_6	*Lewinella nigricans*	NR_112673.1	92%
	Haliscomenobacter hydrossis	NR_074420.1	91%
OTU_3	*Thermomonas koreensis*	NR_113982.1, NR_043584.1	100%
	Thermomonas fusca	NR_025577.1	100%

1. Tay JH, Liu QS, Liu Y (2001) Microscopic observation of aerobic granulation in sequential aerobic sludge blanket reactor. J Appl Microbiol 91:168–175.
2. Altschul SF, Gish W, Miller W, Myers EW, Lipman DJ (1990) Basic local alignment search tool. J Mol Biol 215:403–410. doi: 10.1016/S0022-2836(05)80360-2

Supplementary material

Comparison of the bacterial community composition in the granular and the suspended phase of sequencing batch reactors

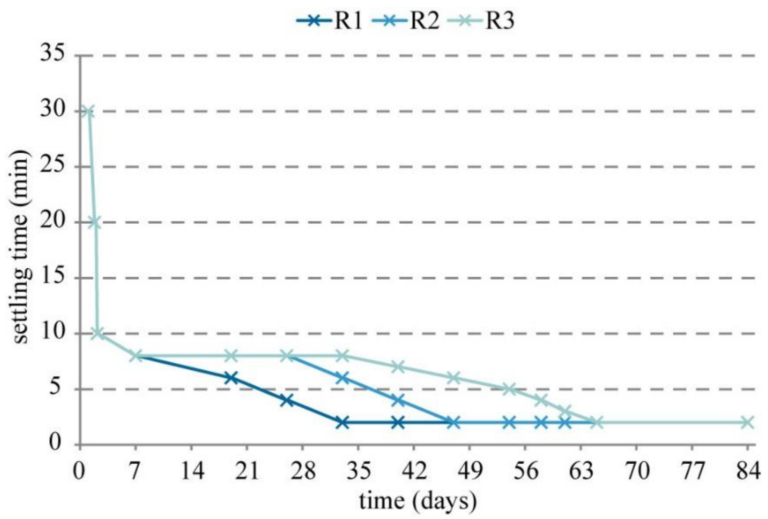

Fig. S1 Gradually decreased settling time

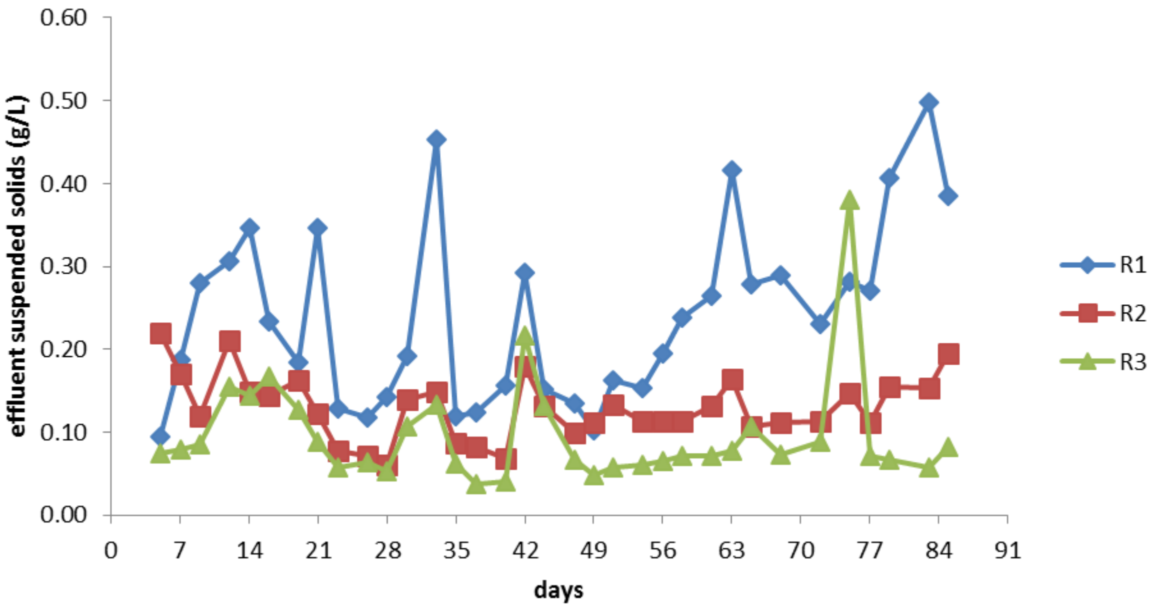

Fig. S2 Suspended solids concentration in the effluent

Comparison of the bacterial community composition in the granular and the suspended phase of sequencing batch reactors

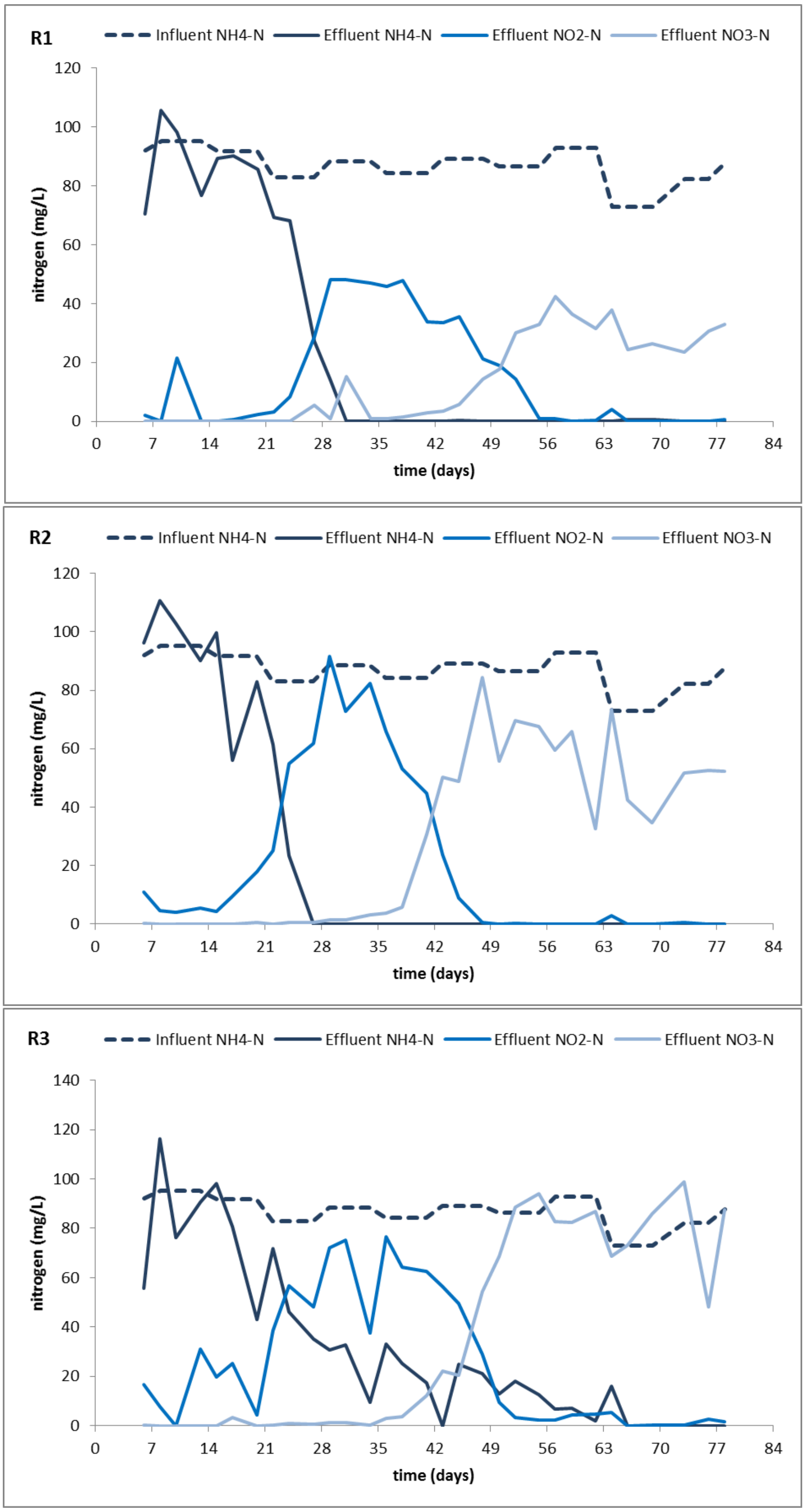

Fig. S3 Ammonium, nitrite and nitrate concentrations in the reactors

Supplementary material

Comparison of the bacterial community composition in the granular and the suspended phase of sequencing batch reactors

(**A**) $p = 2.2 \times 10^{-16}$, $r = 0.803$

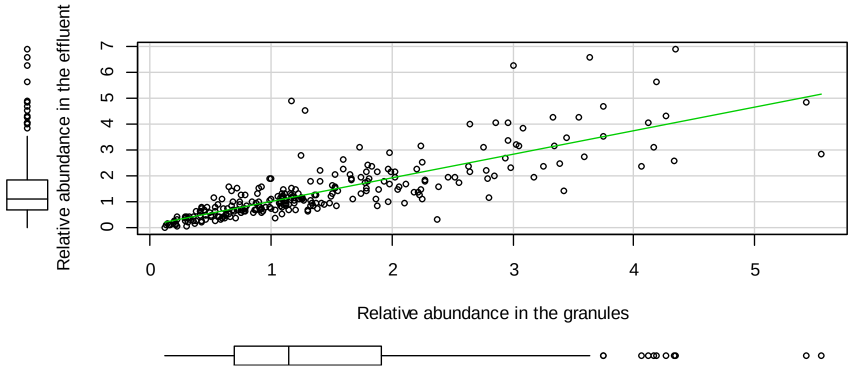

(**C**) $p = 2.2 \times 10^{-16}$, $r = 0.823$

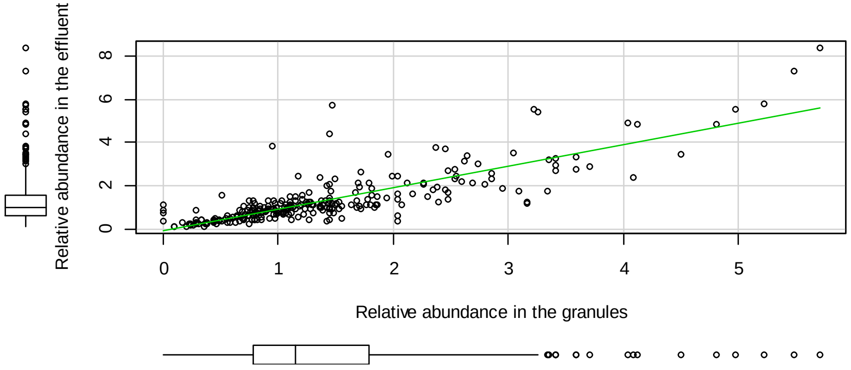

(**B**) $p = 2.2 \times 10^{-16}$, $r = 0.861$

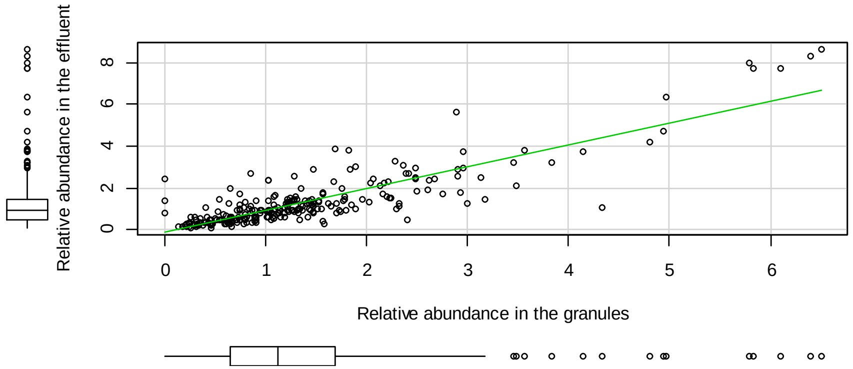

Fig. S4 Correlation analyses of granular and suspended phase samples, (**A**) R1, (**B**) R2 and (**C**) R3. Only the 19 most abundant genera were included in the analysis (these 19 genera add up to 65±11% of the total population in the samples). The relative read abundance of the effluent samples were plotted against the relative read abundance of the granular sludge samples to illustrate the correlation. (Generic R functions were used to perform the Pearson correlation analyses, and the car package [3] was used to visualize the correlation.)

3. Fox, J.; Weisberg, S. *An {R} Companion to Applied Regression*; 2nd ed.; Sage: Thousand Oaks CA, 2011

Supplementary material

Comparison of the bacterial community composition in the granular and the suspended phase of sequencing batch reactors

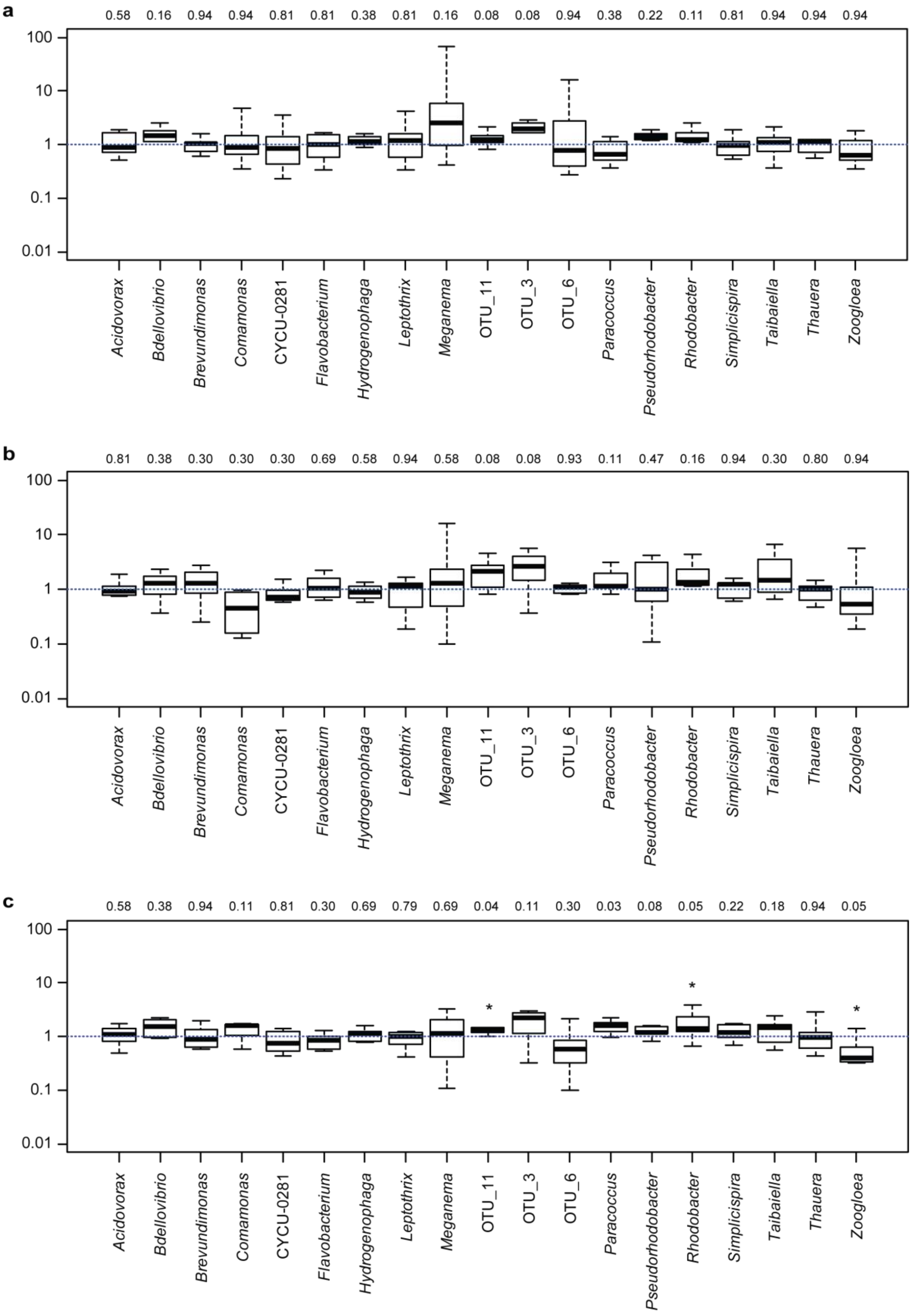

Fig. S5 Boxplots of the retention ratios during start-up (weeks 1-6) in R1 (**a**), R2 (**b**) an R3 (**c**). Values significantly different from 1 are marked with asterisk (p-values are shown above the plots)

Supplementary material

Comparison of the bacterial community composition in the granular and the suspended phase of sequencing batch reactors

A **B**

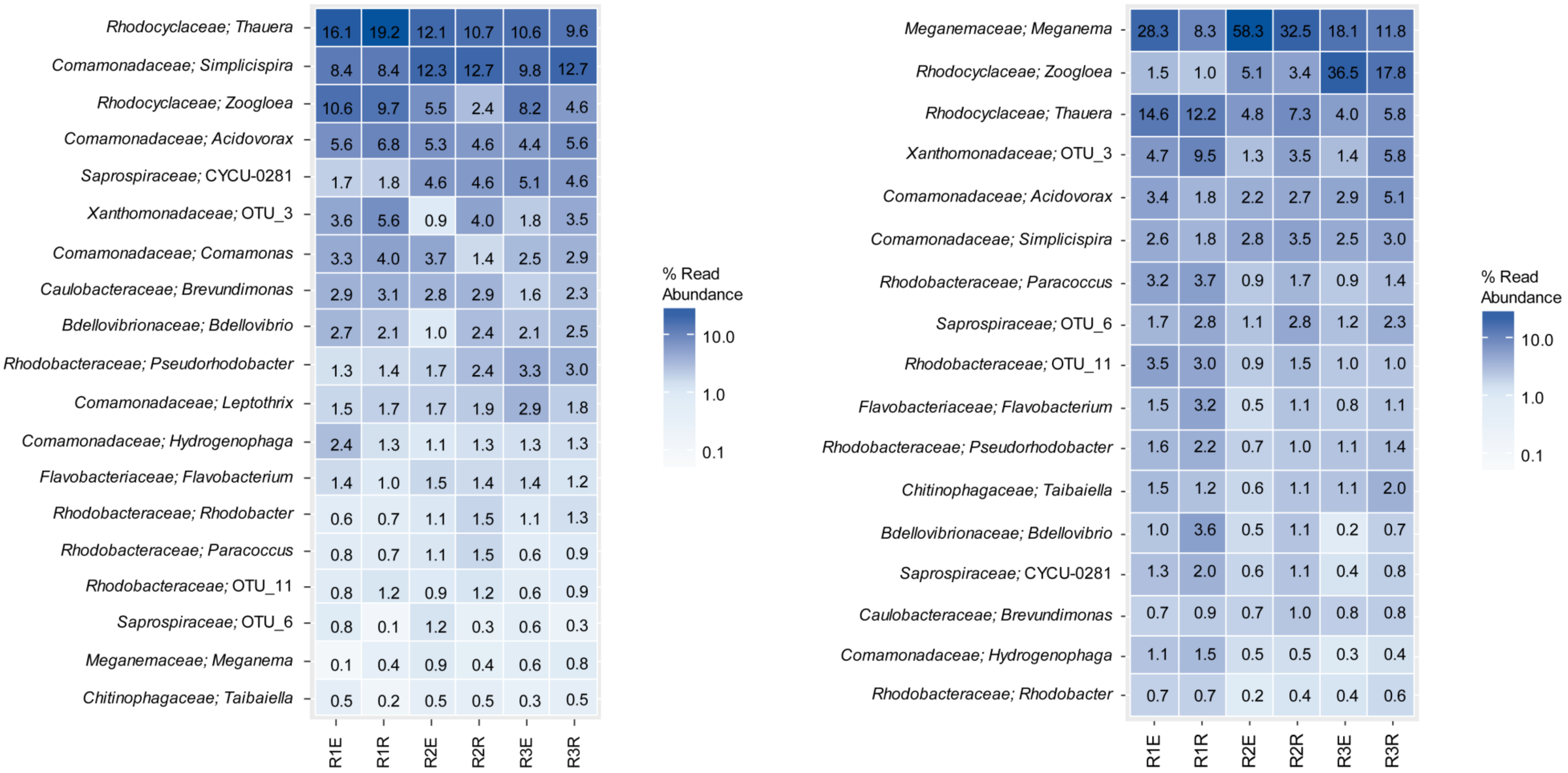

.

Fig. S6 The average relative read abundances of the most common genera during the start-up (**A**) and steady-state (**B**) period. R1E, R2E, R3E: effluent samples (suspended phase); R1R, R2R, R3R: samples from the reactor content (granular phase)

Supplementary material

Comparison of the bacterial community composition in the granular and the suspended phase of sequencing batch reactors

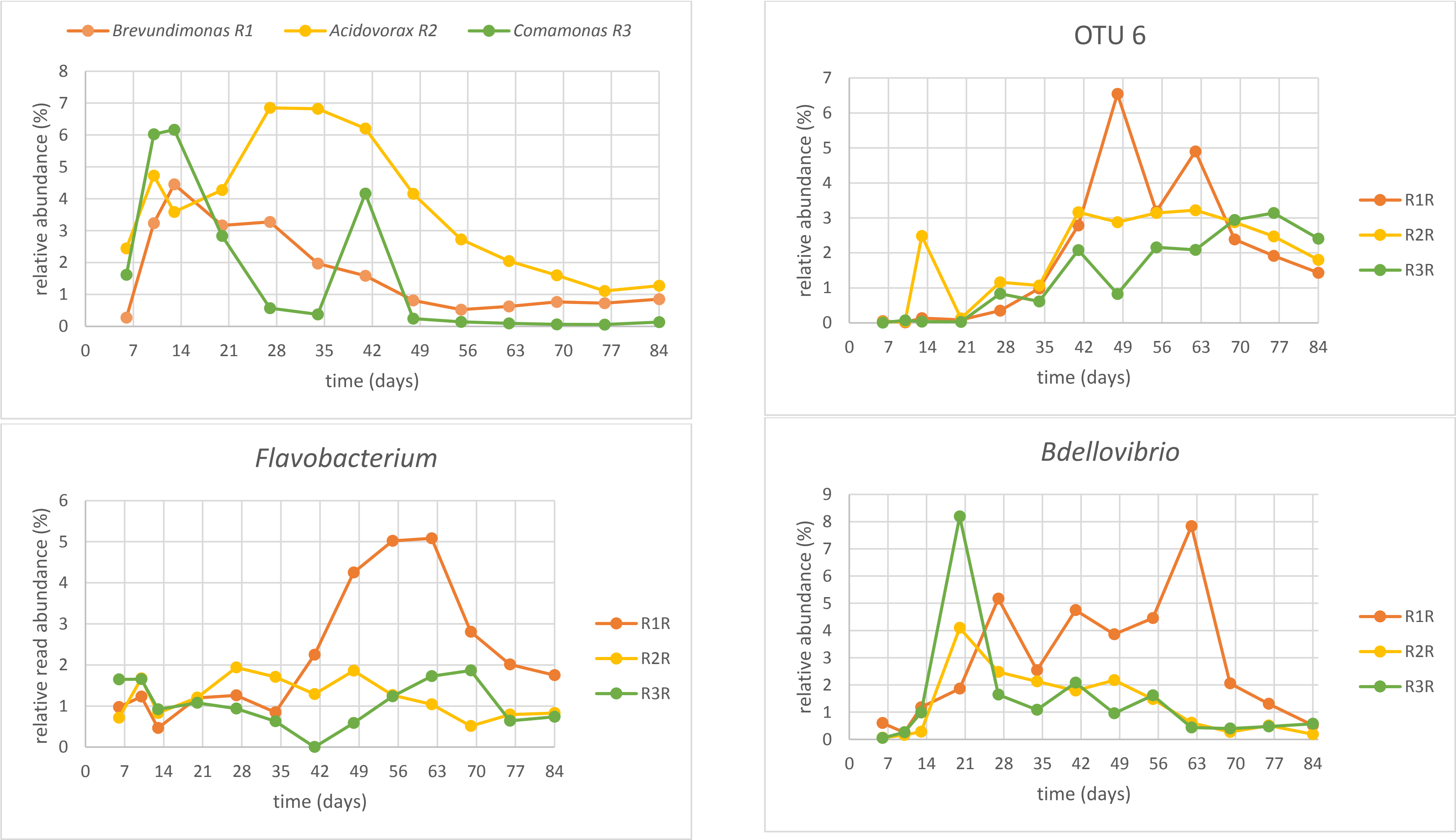

Fig. S7 Temporal variation in the relative read abundance of certain genera
